# The Effects of Vaginal Virome on Women’s Health: A Scoping Review

**DOI:** 10.7759/cureus.97876

**Published:** 2025-11-26

**Authors:** Sara Darwish, Nicole Razdolsky, Kylie Ditty, Andrea Torres, Steven Mathew, Sara Ali, Ayesha Izhar, Jowana Ghazzawi, Christopher Phillips, Amberlynn Braun, Joshua M Costin

**Affiliations:** 1 Medicine, Nova Southeastern University Dr. Kiran C. Patel College of Osteopathic Medicine, Fort Lauderdale, USA; 2 Osteopathic Medicine, Nova Southeastern University Dr. Kiran C. Patel College of Osteopathic Medicine, Fort Lauderdale, USA; 3 Department of Medical Education, Nova Southeastern University Dr. Kiran C. Patel College of Allopathic Medicine, Fort Lauderdale, USA

**Keywords:** human microbiome, vaginal, vaginal virome, virome, women’s health

## Abstract

While research on the vaginal microbiome (VMB) has focused mainly on its bacteriome, the role of viral communities remains largely understudied. Given the vaginal virome's potential influence on disease susceptibility and progression, understanding its exact role in different health outcomes is crucial. The objective of this scoping review was to analyze studies on the vaginal virome's influence on women's health, the virome's interactions with other microbes, assess the limitations of these studies, and highlight gaps to guide future studies and treatments in the field of gynecological health. The review was conducted using previously gathered literature on the impact of the vaginal virome among reproductive-aged women in developed countries. Articles were sourced from Ovid MEDLINE, Embase, and World of Science using the following search string: vaginal virome AND reproductive health OR infections OR pregnancy outcomes. Articles were screened using the PCC framework as follows: influence of the vaginal virome on women’s health (Concept) in women aged 18 and older (Population) in developed countries (Context). Of the 352 articles originally identified, 10 studies met the inclusion criteria and were utilized in this scoping review.

The vaginal virome contains a diverse array of viruses and bacteriophages that impact human papillomavirus (HPV) infection persistence and subsequent progression to cervical cancer, pregnancy outcomes, and the inflammatory response. In bacterial vaginosis, reductions in *Lactobacillus*-targeting bacteriophages have the potential to contribute to dysbiosis. Hormonal fluctuations throughout the menstrual cycle influence both bacterial and viral populations, potentially affecting infection susceptibility and overall vaginal health. During pregnancy, higher viral diversity is associated with preterm birth risk, and immune modulation by the virome may impact susceptibility to infections like HPV. Analysis of the included studies indicates the need for continued investigation of the manner in which the vaginal virome interacts with other aspects of the microenvironment to influence women’s health. This scoping review highlights the new and emerging role of the vaginal virome in women's health, highlighting its relationship with other microbes and clinical significance. These findings underscore the importance of the vaginal virome in reproductive health, suggesting that further research is needed to better understand its role and guide effective prevention and treatment strategies.

## Introduction and background

The human body hosts diverse populations of microbes that play critical roles in maintaining health, with dysbiosis often resulting in disease [1]. While the gut microbiome has been extensively studied, recent research has begun to recognize the pivotal role the vaginal microbiome (VMB) has on women’s health [2]. These microbial communities influence numerous aspects of well-being, including protection against infections, reproductive health, and inflammatory processes [3].

VMB is dynamic and comprises bacteria, viruses, fungi, archaea, and eukaryotes [4]. These specific niches of microbes colonize areas within and outside our bodies, where they hold specific metabolic and immunologic functions [1]. It is important to consider the impact of oncoviruses on community state types (CSTs), which are distinct compositions of the VMB that classify the microbiota based on dominant bacterial species [5]. CSTs are classified into five categories: CST I, dominated by *Lactobacillus crispatus* (*L*. *crispatus)*, CST II by *L*. *gasseri*, CST III by *L. iners*, CST V by *L. jensenii*, and CST IV, which is the most diverse category, comprised of anaerobic bacteria such as *Gardnerella*. CSTs dominated by *Lactobacillus* species (I, II, III, and V) produce lactic acid, resulting in lower vaginal pH, minimizing potential pathogenic growth of bacteria and stabilizing the microbiome [3,5]. This illustrates one of the key protective functions of the VMB possesses. Studies have demonstrated that the composition of the vaginal microbiota varies among different groups. Notably, studies have observed that women of African ancestry have a lower prevalence of *Lactobacillus*-dominated microbiota compared to white women, underlying the importance of genetic and environmental factors in studying the VMB [6]. 

Bacterial vaginosis (BV) is a common form of vaginal dysbiosis characterized by a sudden shift from predominantly lactobacilli species to an overgrowth of anaerobic bacteria such as *Gardnerella vaginalis* and *Mycoplasma hominis* [7,8]. While BV has been traditionally understood to result from bacterial imbalances, recent studies suggest that viruses, particularly bacteriophages, may play a role in its pathogenesis. BV and other forms of vaginal microbiota dysbiosis have been strongly associated with increased susceptibility to sexually transmitted infections/diseases [9]. Other health issues, such as infertility, gestational complications, urinary tract infections, pelvic inflammatory disease, and gynecological cancers, have been linked to dysfunction of the vaginal ecosystem [10-12].

While the bacteriome has been extensively studied, the viral component, termed “virome,” remains understudied, with its contribution to women’s health less understood. An individual's body carries approximately 10^13 ^viral particles, which interact with our immune system, ultimately influencing our microbiomes [13]. The vaginal virome includes eukaryote-infecting viruses and prokaryote-infecting viruses known as bacteriophages [14]. Bacteriophages, which infect and replicate within bacterial hosts, influence the structure and stability of the microbiome. They are typically classified as either lytic or lysogenic. Lytic phages destroy the bacterial host by rupturing the cell wall to release new viral particles, whereas lysogenic phages incorporate their genetic material into the host genome, enabling them to persist and replicate along with the bacterial cell. While bacteriophages can result in imbalances, their impact is more multifaceted. Certain bacteriophages, especially those linked to bacteria found in healthy vaginal environments, may contribute to the maintenance of a balanced vaginal ecosystem [15]. Understanding the mechanism of these viruses is essential when evaluating the influence they have on vaginal microbial health.

The vaginal microbiome’s various components significantly influence women’s health. To fully comprehend the extent of these effects, it is imperative to examine the role that the virome has in relation to the other elements of the vaginal microbiome. Our scoping review aims to address the following question: “How does the vaginal virome play a role in 18+ year old women’s health in developed countries?”

## Review

Methods

This review adhered strictly to the Preferred Reporting Items for Systematic Reviews and Meta-Analyses (PRISMA) guidelines, with all inclusion criteria established before the study began.

Eligibility Criteria

The PCC framework was used to guide this scoping review: the Population (P) consisted of women aged 18 and older; the Concept (C) encompassed studies examining the influence of the vaginal virome on women’s health; and the Context (C) focused on women living in developed countries. Based on these parameters, we established inclusion criteria that captured studies addressing the vaginal virome, its effects on reproductive health, and related infections within developed countries with the goal of identifying a population that has similar environmental inputs. Exclusion criteria included articles not published in English and articles studying mental health.

Search Strategy

A preliminary search of MEDLINE, the Cochrane Database of Systematic Reviews, PROSPERO, and Joanna Briggs Institute (JBI) Evidence Synthesis revealed that there are no current or ongoing systematic reviews on this topic. Articles included in this scoping review will encompass experimental, analytical, observational studies, and case-control studies. The proposed scoping review was conducted in accordance with the JBI methodology for scoping reviews. An initial search was performed in EMBASE on 9/11/2024, using keywords drawn from relevant article titles and abstracts to construct the search strategy. Additional independent searches were conducted by the authors in OVID MEDLINE and Web of Science on 9/12/2024 using the terms “virome” AND “vagina*” AND “reproductive health” OR “infertility” OR “prematurity” OR “pregnancy complications” OR “infection” OR “neoplasm” OR “tumor” OR “cancer.” All review articles were excluded from the final results. 

Article Selection Process

Database search results were imported into Rayyan.io, where duplicates were deleted. Articles underwent two rounds of selection. The first round consisted of 10 authors previewing titles and abstracts against the inclusion criteria, and they were included when all authors agreed. In the second stage, two authors assessed the full text of each article for eligibility, with a third author available to resolve any disagreements. The selection process was documented using the PRISMA extension for Scoping Reviews (PRISMA-ScR) flow diagram. Reasons for excluding studies that did not meet the criteria were recorded and reported. Any misunderstandings among reviewers during screening were addressed through open communication or by involving additional team members.

Critical Appraisal

The articles selected following the Tier II review process were subject to further screening using the JBI Critical Appraisal Tools. This tool functions to avoid research bias and comprises individualized checklists used to assess the quality of each article. This process is crucial to prevent inaccurate results, and it ensures that valid and reliable articles are included in the overall scoping review. Altogether, the critical appraisal process involved two researchers who were assigned to analyze each article blindly and use the appropriate checklist based on the type of research study conducted. Following this, the researchers compared their critical appraisal scores, and each article was thoroughly discussed. Articles with scores less than 50% were classified as high risk of bias, scores between 50% and 70% were considered moderate risk of bias, and scores above 70% were categorized as low risk of bias.

Synthesis of the Results

An initial data summary table was created by extracting key findings from each included study, highlighting correlations between vaginal virome characteristics and women’s health or reproductive outcomes. Using these details, articles were categorized into one of three groups: (1) negative outcomes reported (i.e., higher viral diversity associated with adverse pregnancy outcomes or increased genital inflammation), (2) positive outcomes reported (i.e, *Lactobacillus* dominance correlated with microbial stability and improved reproductive health), or (3) no correlation identified (i.e specific viral presence unrelated to observed clinical outcomes). Studies were included in the relationship mapping only if a domain was correlated with health-related outcomes.

Results

**Figure 1:**
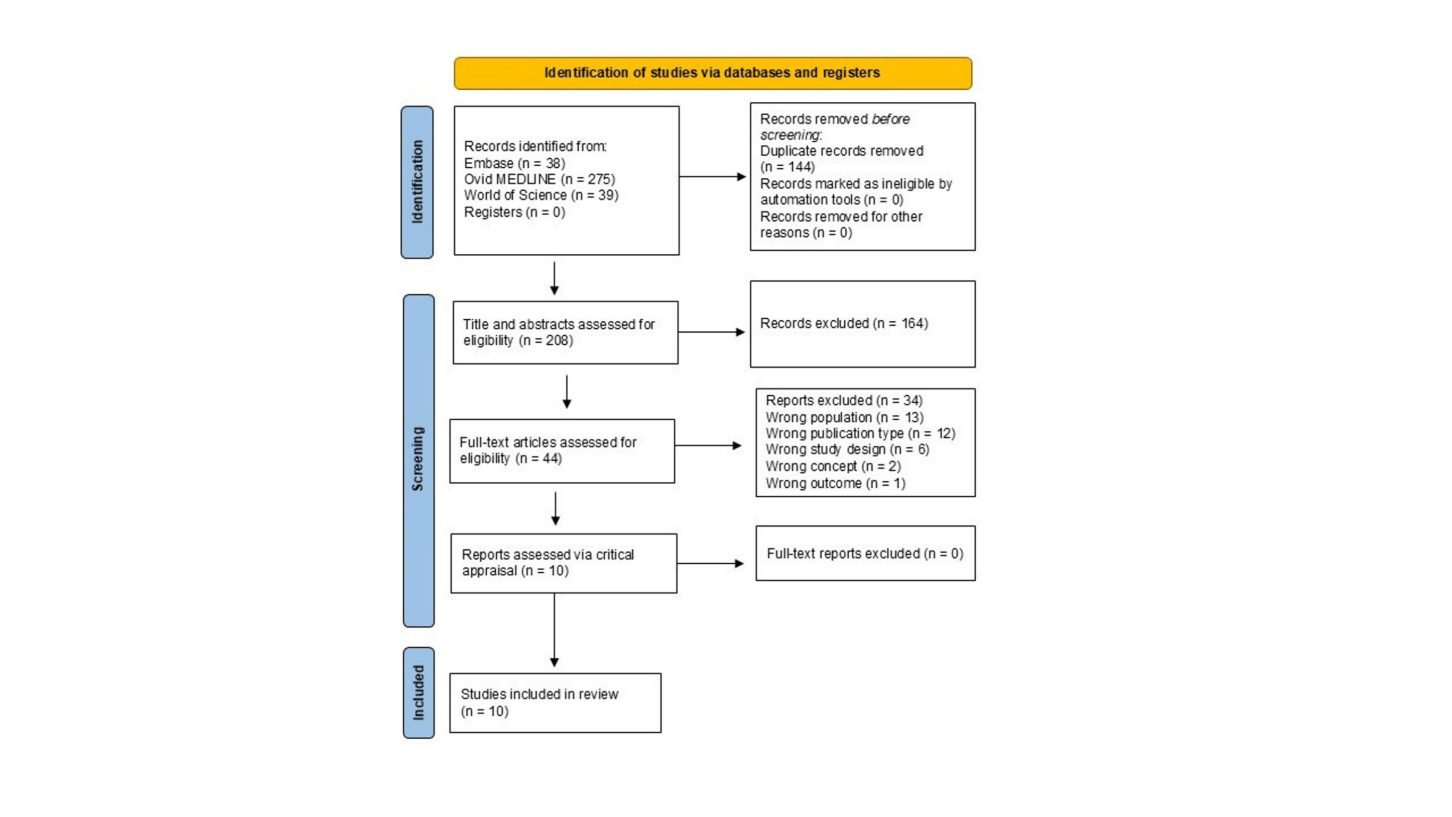
PRISMA-ScR flow chart depicting the article selection process PRISMA-ScR: Preferred Reporting Items for Systematic Review and Meta-analyses extension for Scoping Review

The initial search identified a total of 208 citations through database searches. After removing the duplicates and screening titles and abstracts against predefined inclusion criteria, 10 articles were included in this review. The selection process is outlined in the PRISMA flowchart (Figure 1). All 10 included articles underwent quality appraisal using JBI Critical Appraisal Checklists. Of the selected studies, nine were observational studies, including cross-sectional or prospective exploratory studies [5,16-23] and one was a case-control study [24]. Six of the 10 studies were rated as having a moderate risk of bias [17-18,20-22,24], while four were assessed as low risk [5,16,19,23]. The majority of studies examined associations between the vaginal virome and reproductive outcomes such as BV, cervical cancer, HPV persistence, and preterm birth [5,16-18,23]. Two studies focused on the relationship between the virome and in vitro fertilization (IVF) outcomes [16,20]. Two studies focused on the vaginal virome composition, its interaction with the local immune environment. One study focused on the changes of the vaginal virome across phases of the menstrual cycle [22]. Data from these studies were synthesized into a summary table and were organized into two main categories: (1) virome composition and (2) women’s health and impact on reproductive outcomes (Table 1).

**Table 1:** Summary of the included articles BV: bacterial vaginosis; IVF: in-vitro fertilization; rCCA: regularized canonical correlation analysis; LSIL: low-grade squamous intraepithelial lesions; HSIL: high-grade squamous intraepithelial lesions; CC: cervical cancer; rRNA: ribosomal RNA; PCR: polymerase chain reaction; HPV: human papillomavirus; CIN: carcinoma in-situ; CST: community state type; TTV: torque tenovirus; qPCR: quantitative polymerase chain reaction; MMP: metalloproteinase

Authors	Purpose	Study design	Study population	Methods	Limitations	Key findings
Virome composition						
Jakobsen et al. [16]	Assess vaginal DNA virome and investigate its association with the bacterial community and involvement with BV	Cross-sectional study	48 healthy women of reproductive age undergoing IVF for non-female factor infertility	Metagenomic sequencing of purified virus-like particles from vaginal swabs. Using Bray-Curtis dissimilarity metrics, comparison of the viral community composition was conducted. 16S rRNA gene amplification profiling, quantitative PCR, and rCCA were used to identify the bacterial component of the vaginal microbiome	Limitations include the focus on relative abundance instead of absolute abundance due to viral metagenome sequencing limitations; small sample size; lack of longitudinal sampling; and focus on DNA fraction, excluding the influence of RNA viruses	The vaginal DNA virome is strongly correlated to the bacterial component of the vaginal microbiome and involvement in BV. There was a strong variation in the composition of both prokaryotic and eukaryotic viral communities between BV-negative and BV-positive samples. Strong correlations were found between specific bacteria and bacteriophages (vOTUs), which may influence the composition and stability of the vaginal bacterial community
Kaelin et al. [21]	To investigate the relationship between the cervicovaginal DNA virome and factors that play a role in HPV persistence and progression to cervical cancer, such as the local microenvironment, cervicovaginal microbiota, and genital inflammation	Observational, prospective, exploratory study	23 premenopausal, non-pregnant women	The first vaginal swabs were collected and used for HPV genotyping and 16S rRNA sequencing. The second vaginal swabs were collected and used to assess vaginal pH. DNA was isolated and amplified using PCR. A multiplex cytokine assay was performed to determine genital inflammation status. Bacteriophage analyses were done using BLASTx and VirSorter. ATV qPCR was used to quantify anellovirus load	Small sample size	Genital inflammation is associated with an altered cervicovaginal virome; there is an association between decreased virome richness and alpha diversity and increased levels of *Anelloviridae*. HPV infection is not associated with genital inflammation; *Lactobacillus* bacteriophage contigs are associated with *Lactobacillus* abundance; bacteria-bacteriophage trans-kingdom interactions are associated with genital inflammation and showed interactions with bacterial vaginosis-associated bacteria, including *Gardnerella*, *Prevotella*, and *Sneathia*
Du et al. [22]	Evaluate differences and trends in the microbiome in the mid-vagina and cervical orifice in healthy women throughout the menstrual cycle (follicular, periovulatory, and luteal phases)	Longitudinal observational study	11 non-pregnant reproductive-age (age 20 to 30) Chinese women	Vaginal microecology was done to ensure all subjects’ vaginas were healthy before participation in the study	Small sample size; only women aged 20 to 30 were included, thus results cannot be generalized to women of reproductive age outside of this range	No significant difference in microbiome composition between the mid-vagina and cervical orifice during the corresponding cycle; healthy women living in different regions have significantly different vaginal microbiomes; throughout the menstrual cycle, the amount of *Lactobacillus* increased, and the amount of *Acinetobacter* decreased
Women’s health and impact on reproductive outcomes						
Wylie et al. [24]	Examine the vaginal eukaryotic DNA virome in a cohort of pregnant females and the association between vaginal community characteristics and preterm birth	Case-control study within a prospective longitudinal cohort	60 pregnant women, predominantly (65%) African American	Vaginal swabs were obtained during each trimester. Bacterial communities were characterized by 16S ribosomal RNA gene sequencing. DNA was isolated and prepared using high-throughput sequencing with ViroCap, targeting the eukaryotic viral community. Viral communities were analyzed, including their association with the bacterial community	Limitations include a smaller number of swabs during the first trimester; no distinction between viral exposure from active replication; focus on eukaryotic viral DNA viruses	Viral families are present in the vaginas of pregnant women with both full-term and preterm birth outcomes. High viral richness was associated with preterm birth. Higher diversity of bacterial and viral components was associated with the highest risk of preterm birth in the first trimester. No single virus was associated with preterm birth
Li et al. [17]	Examine the virome in women with different levels of cervical lesions and the association with cervical disease status	Cross-sectional study	161 women: 53 with normal cervical tissue, 46 with LSIL, 36 with HSIL, 26 with CC	Vaginal swabs were collected and further assessed via viral metagenomic sequencing and 16S rRNA sequencing to profile the viral and bacterial microbiome. Viral and bacterial communities and their correlation to cervical disease status were assessed. Quantitative PCR was used to assess viral loads	Limitations include study design, lack of longitudinal samples to track virome over time, limited cohort size restricted to a single cervical clinic, and focus on eukaryotic viral DNA viruses	Eukaryotic viruses such as anelloviruses and papillomaviruses were found in higher abundance and richness in women with LSIL and CC. These viruses were also found in *Lactobacillus*-depleted vaginal microbiomes. This suggests their influence on host physiology and the potential of anellovirus to be used as a biomarker for the prediction and prognosis of cervical diseases
Sasivimolrattana et al. [18]	Examine virome composition in HPV-infected cervix and its association with viral diversity and cervical disease	Observational study	35 patients ranging from 23-50 years old with blood with HPV infection, further classified as CIN1 and CIN2/3	Cervical swab samples were collected, and virome capture sequencing and metagenomic sequencing were done to analyze the viral diversity and composition of HPV-infected cervixes	Limitations include a limited sample size and a lack of diverse control groups	HPV was the most dominant virus in most cervical samples. A higher amount of HPV correlated with lower amounts of other human viruses. HPV co-infection with other viruses might act as a cofactor in HPV-related cancers
Campisciano et al. [5]	Evaluate the impact of oncoviruses on vaginal microbiome and immune response in women of reproductive age	Observational study	29 (20-40 years old) Caucasian immunocompetent women of reproductive age who tested positive for viral infection	Vaginal swabs collected. DNA was extracted using the NucliSENS easyMAG system and amplified. Metagenomic sequencing and multiplex PCR were used for oncogenic viruses. Cytokine and chemokine concentrations were assessed via quantitative cytokine assay	Limitation includes a small study cohort	Vaginal swabs were divided into 4 vaginal CSTs based on predominant *Lactobacillus* species. CST I (*Lactobacillus crispatus*- dominated) was linked to efficient viral clearance, while CST III-IV (*Lactobacillus iners* and depleted), respectively, were associated with persistent HPV and polyomavirus infections. Infected samples varied in immune response, with reduced presence of antiviral cytokines
Tozetto-Mendoza et al. [19]	Investigate the presence of TTV in vaginal secretions during pregnancy and the postpartum period, and its influence on the vaginal microbiome and immune markers	Observational study	121 pregnant women from New York City who delivered at different terms/entered the postpartum period	Vaginal swab samples were collected, DNA was extracted, and amplified via qPCR. Concentrations of matrix MMP-8 and lactic acid were determined via ELISA kits. Vaginal microbiome composition was analyzed	Limitations include a lack of maternal blood samples to determine the origin of TTV, whether from vaginal or systemic sources. Cord blood was not available to determine the transplacental passage of TTV from mother to baby. Lack of analysis of additional viruses; one geographic population	TTV was detected in ~40% of the patients, with the viral titer higher in the postpartum period. Women positive for TTV in their first trimester were found to have a lower gestational age. Higher levels of MM8 were associated with the presence of TTV. *L. crispatus* dominance and high lactic acid levels were associated with lower TTV prevalence
Eskew et al. [20]	Evaluate the association between the vaginal virome, antibiotic exposure, and IVF outcomes	Planned a priori prospective exploratory study	26 subfertile women between 18 to 43 years of age who were undergoing their first IVF cycle with a fresh embryo transfer	A mid-vaginal swab was obtained immediately before embryo transfer to analyze the effect of prophylactic azithromycin on the outcome of clinical pregnancy via IVF. Two subject groups were included: the azithromycin group and the no-azithromycin group. DNA was extracted using the QIAmp BiOstic Bacteremia DNA Kit	Small sample size; lack of adjusted P-values to correct for multiple testing; inability to rule out the potential confounding effect of cefazolin prophylaxis that subjects received at the time of egg retrieval; no control group who did not receive prophylactic cefazolin; varying time between antibiotic exposure and collection; only one time-point during the IVF cycle was observed; DNA sequencing does not distinguish between viral exposure and active replication; only eukaryotic DNA viruses were assessed; lack of RNA sequencing excludes RNA viruses from the study; population was predominantly white and non-Hispanic; only fresh embryo transfers were included, so data is not generalizable to frozen embryo transfers	No significant differences in IVF outcomes between the azithromycin group and the no- no-azithromycin group; no association between viral diversity and clinical pregnancy; the azithromycin group had a higher diversity of herpesviruses and ⍺papillomaviruses; within the azithromycin group, the women who did not achieve clinical pregnancy were found to have a higher viral diversity
Stout et al. [23]	Determine how pregnancy impacts host-virus dynamics; expand upon a previous study, which showed that higher viral diversity in the vagina during pregnancy is associated with Black race and preterm birth; test if higher diversity and viral copy numbers would also be associated with Black race and preterm birth	Retrospective cohort study	23 pregnant patients (11 term and 12 preterm)	Longitudinally collected plasma samples from pregnant patients were evaluated using metagenomic sequencing with ViroCap enrichment. Nucleic acid was sequenced and analyzed, and qPCR was used to quantify anellovirus genome copy numbers	Small sample size; only two racial groups present (black and white)	The Black race is associated with higher viral richness in maternal blood samples; no associations were found between viral richness, preterm birth, or the trimester of sampling. Higher anellovirus positivity, but not copy numbers, was found to be associated with Black race; anellovirus positivity and copy numbers were higher in the preterm birth group

Virome Composition

Across eight studies, the vaginal virome consists of a diverse array of viruses, including human eukaryotic viruses, DNA viruses, RNA viruses, non-human primate-associated viruses, and bacteriophages [5,18-22,24]. While viruses not known to infect humans have been identified in samples, this scoping review focuses on human-associated viruses relevant to the vaginal and cervical microbiome [18,22]. VMB plays a crucial role in women’s reproductive health, with the bacterium *Lactobacillus* being a key factor in maintaining a healthy microbiome [3]. The most commonly identified eukaryotic DNA and RNA viruses belong to *Herpesviridae*, *Papillomaviridae*, *Polyomaviridae*, *Poxviridae*, *Adenoviridae*, *Anelloviridae*, *Hepadnaviridae*, and *Retroviridae* families detected in vaginal or cervicovaginal samples [17,24].

The *Herpesviridae* family, particularly cytomegalovirus and herpes simplex virus, was frequently detected in the vaginal samples and is known for their ability to establish latent infections [20]. Oncogenic strains of the *Papillomaviridae* family, including high-risk HPV types, were identified in multiple studies, highlighting their potential impact on reproductive outcomes [5,18,24]. Additionally, *Anelloviridae* viruses, specifically, torque teno viruses (TTV), were found in high concentrations and were linked to dysbiosis of the VMB [19]. Bacteriophages, including *Siphovirdae*, *Myoviridae*, *Podoviridae*, *Herelleviridae*, *Microviridae*, and *Inoviridae,* were identified in cervicovaginal samples, collectively playing a crucial role in maintaining microbial balance, while also driving dysbiosis under certain conditions [17,21].

Women’s Health and Impact on Reproductive Outcomes

Across two studies, the relationship between the vaginal virome, BV, and microbiome fluctuations across the menstrual cycle was investigated to determine how viral and bacterial stability influence overall vaginal health [16, 22]. Greater viral diversity in BV-positive samples was observed, with BV-associated bacteria (*Gardnerella* and *Moraxella*) linked to fewer phages targeting *Lactobacillus* species, indicating a shift in microbial stability [16]. Additionally, *Herpesvirales* and *Papillomavirdae* are present in higher numbers in BV-positive samples compared to BV-negative samples, suggesting the possible influence eukaryotic viruses have in disrupting the homeostasis of the VMB [16]. During the periovulatory phase (period B), a decline in *Lactobacillus* dominance coincided with increased viral diversity, suggesting that hormonal fluctuations create an environment more susceptible to viral invasion and infection [22]. The role of the virome composition in cervical HPV infections was investigated by one study, which found that higher HPV abundance was associated with lower overall cervical viral diversity [18]. At the same time, there was greater cervical viral diversity in the non-HPV-dominated (NHD) group when compared to the HPV-dominated (HD) group.

Two studies aimed to establish an association between the abundance of viruses in the VMB and the inflammatory response [5,21]. The role of oncoviruses such as *Polyomaviridae*, *Papillomaviridae*, and *Herpesviridae* on host immune response and vaginal CSTs was investigated. There are five CSTs, and CSTs I, II, III, and IV were evaluated in this study, along with a mixed CST category [5]. The presence of *Polyomaviridae* and *Papillomaviridae* caused an increase in *Lactobacillus crispatus* in CST I and a decrease in both *Prevotella timonensis* and *Sneathia sanguinegens* in CST IV. The altered immune response of CST II and III indicated reduced antiviral capacity. CST II and III both showed increased pro-inflammatory cytokines, decreased anti-inflammatory cytokines, and decreased anti-tumor factors. Viral clearance was at a maximum when *L. crispatus* was dominant, as in CST I. The persistent HPV and polyomavirus co-infections found in CST III and IV indicated a dysbiotic state [5].

Three studies investigated how the vaginal virome influences pregnancy outcomes, examining its potential role in microbial stability, viral diversity, and reproductive health risks [20,23-24]. Higher overall viral-bacterial diversity in the first trimester was associated with spontaneous preterm birth; however, when analyzed individually, neither viral nor bacterial diversity alone showed a statistically significant association [24]. Anelloviruses were the most commonly detected viral family (42%), followed by papillomaviruses (40%), but no specific virus or viral group was directly associated with preterm birth [24]. *Papillomaviridae* was the most prevalent viral family at the time of embryo transfer, with other dominant viral families, including *Herpesviridae*, *Polyomaviridae*, and *Anelloviridae,* also present [20]. *Anelloviridae* was the most prevalent virus in maternal plasma, with higher positivity and genome copy numbers associated with preterm birth [23].

Discussion

Principal Findings

This scoping review examines current evidence on the role of the vaginal virome in women's health, highlighting its composition and potential clinical significance. A total of 10 studies were included, covering topics such as the virome’s influence on microbial balance, its association with different gynecological health conditions, and immune response dynamics. Collectively, these studies demonstrate that increased viral diversity and complex virus-bacteria interactions within the vaginal microbiome are linked to BV, cervical cancer progression, adverse pregnancy outcomes, and immune modulation, underscoring the virome’s critical yet largely underexplored role in women's health outcomes.

Role of the Vaginal Virome in Bacterial Vaginosis-Associated Health Risks

The role of the vaginal virome and its interaction with bacterial populations may be important in determining the risk or progression of BV. BV-positive samples show increased viral diversity with larger amounts of both prokaryotic and eukaryotic viral communities compared to BV-negative samples [16]. Research determining whether having BV allows the overgrowth of other viral communities, or whether there are certain viruses that increase susceptibility to BV, is vital in determining what this increased diversity means for women's health. The relative abundance of *Lactobacillus*-targeting bacteriophages was also reduced in the presence of BV-associated bacteria such as *Gardnerella* and *Moraxella*, suggesting the transition to an unstable *Lactobacillus*-depleted microbiome [16]. Thus, a reduction in bacteriophages targeting *Lactobacillus* may simply be a reflection of a decrease in the availability of its host. Conversely, it may contribute to dysbiosis by allowing the overgrowth of BV-causing bacteria, presumably from killing too many *Lactobacilli* under certain conditions. This underscores the role of bacteriophages in maintaining microbial balance, warranting further investigation into the virome's role in BV susceptibility and overall vaginal health.

Additionally, studies have highlighted the increased presence of *Herpesvirales*, *Papillomaviridae*, and *Anelloviridae* in BV-positive samples, supporting BV as a potential contributor to susceptibility to concurrent viral infections [16]. It was not noted, however, whether these viruses existed before the diagnosis of BV, or if having BV allowed for an environment more conducive to the survival of these pathogens. Collectively, these associations highlight the importance of virus-bacteria interactions in vaginal health, particularly with respect to the development and maintenance of BV. Research may be pivotal in creating targeted therapies that help maintain microbial balance with the goal of preventing the adverse health effects associated with BV.

Menstrual Cycle-Driven Microbiome and Virome Fluctuations

The VMB shifts throughout the menstrual cycle, with hormonal changes possibly influencing bacterial and viral populations [22]. Throughout the cycle, the relative abundance of *Acinetobacter* decreases while the relative abundance of *Lactobacillus* increases [22]. This dominance is thought to occur to prepare for a newborn, as *Lactobacillus* helps infants digest milk, their primary food source in the first few months of life [25]. Interestingly, human alphaherpesvirus 1 levels have also been shown to be increased during the periovulatory phase [22]. These variations may suggest that changing hormones throughout the menstrual cycle impact levels of both viruses and bacteria in the vagina, potentially contributing to effects on infection susceptibility and overall vaginal health. The detection of human alphaherpesvirus 1 during the periovulatory phase also suggests that some viruses may predominate or become reactivated during specific phases of the menstrual cycle. On the other hand, vaginal microbe composition remains consistent between the mid-vagina and cervical orifice during the periovulatory and luteal phases, indicating that susceptibility to hormonal fluctuations may not occur throughout all of the hormone-sensitive tissues [22].

Vaginal Virome and Pregnancy Outcomes

Multiple studies have suggested a role for the vaginal virome in pregnancy outcomes, but the exact influence of specific members of this viral community is unknown [24]. Pregnant women have a wide variety of viral families with both full-term and preterm birth outcomes; however, preterm birth has been associated with high viral diversity [24]. Furthermore, increased viral and bacterial diversity during the first trimester was associated with an increased risk of preterm birth [24]. However, when viral and bacterial diversity were evaluated separately, neither had a statistically significant association. This suggests that interactions between the two are affecting birth outcomes. On the other hand, higher positivity rates and copy numbers of *Anelloviridae* were identified in a different study that were associated with an increased risk of preterm birth [22]. It is important to understand if observed changes in the virome are driving pregnancy outcomes or if they are merely a reflection of fundamental physiological changes. Longitudinal randomized controlled studies may be needed to establish these interactions and guide strategies for improving maternal-fetal health.

Virome and the Immune Response

The vaginal virome modulates the inflammatory response component of the general immune response [5]. Infection by HPV and polyomavirus promotes the colonization of *Prevotella timonensis* and *Sneathia sanguinegens* in the vaginal mucosal environment by suppressing the pro-inflammatory response through decreasing the concentration of the pro-inflammatory cytokines IL-7 and IL-15, as well as the anti-inflammatory cytokine IL-9 in CSTs II and III [5]. This effect may be specific to CST II and III due to their less stable, *Lactobacillus*-poor environments, which are more susceptible to immune modulation by viral infection. These findings emphasize the dynamic nature of the pathways involved in the immune response and how they may contribute to dysbiosis of the VMB.

In addition to modulation of the inflammatory response, the vaginal virome is also capable of impacting the immune response via its interaction with different microbial entities [19]. Annelloviruses, specifically, the TTVs, were linked with genital inflammation through their ability to enhance pro-inflammatory cytokines and increased diversity of viral contigs, which are continuous DNA sequences representative of viral genomes, associated with inflammation [19]. This increases the vaginal environment’s susceptibility to cervical carcinoma, HIV, and other sexually transmitted diseases [19]. The correlation of annelloviruses with the appearance of inflammation-associated diseases indicates that further investigation must be conducted to uncover therapies that target the vaginal virome to mitigate the development of inflammation.

Furthermore, bacterial species in the VMB may also contribute to inflammation in the vaginal environment [5]. CSTs containing high quantities of *L. crispatus* were associated with increased clearance of HPV and MCPyVm, while CSTs containing *L. gasseri* or *L. iners* were found to have higher amounts of pro-inflammatory factors and lower amounts of anti-inflammatory and anti-tumor factors [5]. While *Lactobacillus* organisms typically protect the vagina from invasive pathogens, dominating concentrations of different species yield different effects, which indicates that each species employs different mechanisms in its defense that, in excess, can produce adverse effects. This further reinforces the idea that homeostasis vs. dysbiosis of the vaginal virome plays a role in the health outcomes of women, making it extremely apparent that a homeostatic environment is critical in order to prevent vaginal dysbiosis.

HPV, Cervical Cancer, and the Virome

Studies examining virome composition in HPV-positive and HPV-negative samples suggest that viral diversity may play a role in cervical cancer development [18]. Notably, cervical viral diversity has been observed to be higher in NHD populations compared to HD populations [18]. The greater viral diversity seen in non-HPV populations may suggest that a balanced virome limits the overgrowth of individual viruses. Conversely, HPV persistence may reflect its capacity to suppress or outcompete other viral species. These patterns imply that non-HPV viruses could shape the cervicovaginal environment through mechanisms such as viral interference or immune modulation (e.g., type I interferon), ultimately influencing HPV survival and disease progression [18]. These interactions warrant further investigation into how viral diversity in the cervicovaginal environment can potentially impact cervical carcinogenesis.

Furthermore, the bacterial microbiome may also work together with the virome to affect cervical cancer development. *Lactobacillus* is known to provide a stable environment in the vagina, and its depletion has been linked to greater susceptibility to a range of infections, including viral infections (e.g., HPV), bacterial overgrowth (e.g., *Gardnerella vaginalis*), and fungal pathogens (e.g., *Candida spp.*) [3]. To this end, dysbiosis of the microbiome has been associated with higher levels of eukaryotic viruses, such as anelloviruses and papillomaviruses, in women with low-grade squamous intraepithelial lesions (LSIL) and cervical cancer [17]. Thus, *Lactobacillus* may have a protective role against cervical cancer by preventing virus colonization and/or persistence. Research into this interaction may allow the development of targeted treatment to not only prevent but also possibly treat cervical cancer. It also opens the door to the use of specific microbes, such as *Lactobacillus*, to be used as biomarkers to determine cervical cancer risk and prognosis.

Additionally, *Anelloviridae* are found in higher abundance among individuals with cervical carcinoma, which is also linked to greater disease severity [17]. This highlights the potential use of *Anelloviridae* to serve as a biomarker for predicting quicker disease advancement. *Papillomaviridae*, on the other hand, is more abundant in LSIL than in cervical carcinoma, suggesting a greater presence in earlier disease stages compared to advanced stages [17]. This demonstrates the complexity of viral interactions within the cervicovaginal environment and suggests that different viruses may play unique roles at various stages of cervical cancer progression. While it is uncertain whether the increased presence of *Anelloviridae* and *Papillomaviridae* is causative of LSIL to cervical carcinoma progression, or whether the progression of lesions themselves leads to greater virus abundance, the associations in this study suggest an investigation into these relationships to determine potential causation.

Gaps in the Literature

Many of the existing studies focused on DNA viruses, but the role of RNA viruses in the vaginal virome and their subsequent effect on women’s health is largely unexplored. There are some indications that RNA viruses could have a significant impact on the vaginal virome [16]. RNA-virus targeting therapies or treatments that enhance the defense mechanisms of the vaginal microbiome can be developed.

Another gap in the literature pertains to the effects of hormones on the vaginal virome and how their associated fluctuations impact women’s health. While there was some investigation into how the prevalence of *Lactobacillus* in the vaginal virome increased as the menstrual cycle progressed, there is no evidence detailing the hormonal component of this increased presence [22]. Hormones play a variety of roles, including in immune response and the microbiome makeup. Further research into which specific hormones, at specific levels, and the mechanisms by which they modulate the vaginal virome, could lead to a more complete understanding of how vaginal virome dysbiosis may contribute to adverse health outcomes in women.

Limitations

This review has several methodological limitations that may introduce bias into the research process. Selection bias is possible, as the search relied on specific keywords and was limited to three databases, potentially omitting relevant studies. Additionally, many of the included studies were characterized by small sample sizes, limiting the generalizability of their findings and reducing the extent to which results can be applied to the broader female population.

Finally, limiting the review to females aged 18 and older from developed countries may represent an additional constraint, as it excludes minor populations and women from underdeveloped regions. Pertinent alterations in the vaginal virome may occur before age 18, and disease prevalence can vary substantially across geographic and socioeconomic contexts. Certain diseases are more common in underdeveloped countries but are practically eradicated in more developed countries. Consequently, excluding women from underdeveloped countries may have limited the ability to capture important insights into how the vaginal virome relates to women’s health across diverse settings. Although the goal of research in this area is to generate robust, generalizable findings for the broader female population, these limitations constrain the scope of the current evidence.

## Conclusions

This scoping review reveals that the vaginal virome is a diverse and dynamic component of the microbiome, intricately linked to BV, HPV persistence, cervical cancer progression, immune modulation, and pregnancy outcomes. Increased viral diversity and specific viral families, particularly *Anelloviridae*, *Papillomaviridae*, and *Herpesviridae*, are associated with dysbiosis, inflammation, and adverse reproductive outcomes. However, current evidence is limited by small sample sizes, geographic and demographic homogeneity, and a focus on DNA viruses, leaving the role of RNA viruses and hormonal influences largely unexplored. Broader, longitudinal studies incorporating diverse populations and hormonal profiling are needed to understand how viral-host interactions influence vaginal health over time. Future research should prioritize uncovering causal pathways between viral shifts and gynecologic disease, investigate hormonal regulation of the virome across the menstrual cycle, and explore the diagnostic or therapeutic potential of viral and bacterial biomarkers. Continued research on this topic will enhance our understanding of how vaginal virome dysbiosis contributes to disease development, ultimately serving as a stepping stone for improving health outcomes for women worldwide.
